# Epilepsy protein Efhc1/myoclonin1 is expressed in cells with motile cilia but not in neurons or mitotic apparatuses in brain

**DOI:** 10.1038/s41598-020-79202-4

**Published:** 2020-12-16

**Authors:** Toshimitsu Suzuki, Ikuyo Inoue, Kazuhiro Yamakawa

**Affiliations:** 1 Department of Neurodevelopmental Disorder Genetics, Institute of Brain Science, Nagoya City University Graduate School of Medical Science, 1 Kawasumi, Mizuho-cho, Mizuho-ku, Nagoya, Aichi 467-8601 Japan; 2 Laboratory for Neurogenetics, RIKEN Center for Brain Science, Wako, Saitama 351-0198 Japan

**Keywords:** Diseases, Neurological disorders, Epilepsy

## Abstract

*EFHC1* gene encodes the myoclonin1 protein, also known as Rib72-1. Pathogenic variants in *EFHC1* have been reported in patients with juvenile myoclonic epilepsy (JME). Although several studies of immunohistological investigations reproducibly showed that the myoclonin1 is expressed in cells with flagella and motile cilia such as sperm, trachea and ependymal cells lining the brain ventricles, whether myoclonin1 is also expressed in neurons still remains controversial. Here we investigated myoclonin1 expression using widely-used polyclonal (mRib72-pAb) and self-made monoclonal (6A3-mAb) anti-myoclonin1 antibodies together with *Efhc1* homozygous knock-out (*Efhc1*^−/−^) mice. All of the western blot, immunocytochemical, and immunohistochemical analyses showed that mRib72-pAb crossreacts with several mouse proteins besides myoclonin1, while 6A3-mAb specifically recognized myoclonin1 and detected it only in cells with motile cilia but not in neurons. In dividing cells, mRib72-pAb signals were observed at the midbody (intercellular bridge) and mitotic spindle, but 6A3-mAb did not show any signals at these apparatuses. We further found that the complete elimination of myoclonin1 in *Efhc1*^−/−^ mouse did not critically affect cell division and migration of neurons in cerebral cortex. These results indicate that myoclonin1 is not expressed in neurons, not a regulator of cell division or neuronal migration during cortical development, but expressed in choroid plexus and ependymal cells and suggest that *EFHC1* mutation-dependent JME is a motile ciliopathy.

## Introduction

Heterozygous pathogenic variants in the *EFHC1* (EF-hand domain containing 1) gene have been well described in patients with JME and other types of idiopathic epilepsies^1–8^. As a rare case, a homozygous variant of *EFHC1* has been identified in 2 siblings with intractable epilepsy of infancy in one family^9^. *EFHC1* encodes an approximately 75 kDa non-ion channel protein myoclonin1 that is composed of three consecutive DM10 domains, a motif of unknown function, and one EF-hand calcium-binding motif at the C terminus^1,10^.

Ikeda and colleagues^11^ reported that immunofluorescence imaging and western blot analyses using a rabbit polyclonal antibody raised against mouse myoclonin1 (mRib72-pAb) revealed that the immunosignals were observed in sperm flagella and tracheal motile cilia in mouse but absent in immotile primary cilia of NIH3T3 cultured cells. We originally reported neuronal expression of myoclonin1 in the immunohistochemical analyses of mouse brain using our self-made rabbit polyclonal antibody raised against myoclonin1^1^. However, we subsequently generated *Efhc1*^−/−^ mouse^12^, and by using this mouse as a negative control we found that the immunosignals in neurons obtained by the polyclonal antibody remained in the *Efhc1*^−/−^ mouse and that the immunosignals in neurons were therefore non-specific^10^. We also reported that a new mouse monoclonal antibody raised against myoclonin1 (6A3-mAb) revealed that myoclonin1 was dominantly expressed in fetal choroid plexus epithelial cells, motile cilia of ependymal cells, tracheal motile cilia, and sperm flagella at postnatal stages, but not expressed in progenitors of the developing cortex in the fetus and matured neurons^10^. Consistently to our observations, Conte and colleagues reported that *Efhc1* mRNA was detected in the ependymal cells and choroid plexus, but not in neurons in mouse and rat brains^13^.

Contrarily to our observations of the absence of myoclonin1 in neurons, another group in Belgium reported that the mRib72-pAb immunosignals were observed in neurons of various brain regions (all cortical layers, piriform cortex, hippocampus and cerebellum) and radial glia cells in embryonic cortex, in addition to ependymal cells and choroid plexus in mouse^14^ and in cytoplasm, nuclei, centrosome, mitotic spindle and midbody of cultured cells^14–17^. They further reported that a suppression of *Efhc1* by small hairpin RNAs (shRNAs)-mediated RNA interference (RNAi) in cultured cells or rat embryonic brain caused disruption of mitotic spindle structure, impaired M-phase progression, increase of apoptosis, impaired cell cycle exit of cerebrocortical progenitors, defective radial glia scaffold organization, impaired locomotion of postmitotic neurons, and marked disruption of radial migration^17^. With these results, they proposed that myoclonin1 is a regulator of cell division and neuronal migration during cortical development and that disruption of its function leads to JME^17^.

In order to further investigate the above-mentioned discrepancy for the distribution of myoclonin1, in the present study we carefully re-examined the histological and cytological distributions of myoclonin1 in mouse brain and cultured cells by using the mRib72-pAb and 6A3-mAb antibodies together with the *Efhc1*^−/−^ mouse. Our results show that the mRib72-pAb signals in neurons are non-specific and myoclonin1 is expressed in cells with motile cilia but not in neurons and that *Efhc1*-deficiency causes no apparent abnormalities in cell division, radial glia scaffold organization and apoptosis in brain.

## Results

### The 6A3-mAb specifically detects myoclonin1, but mRib72-pAb non-specifically crossreacts with additional proteins besides myoclonin1

**Figure 1:**
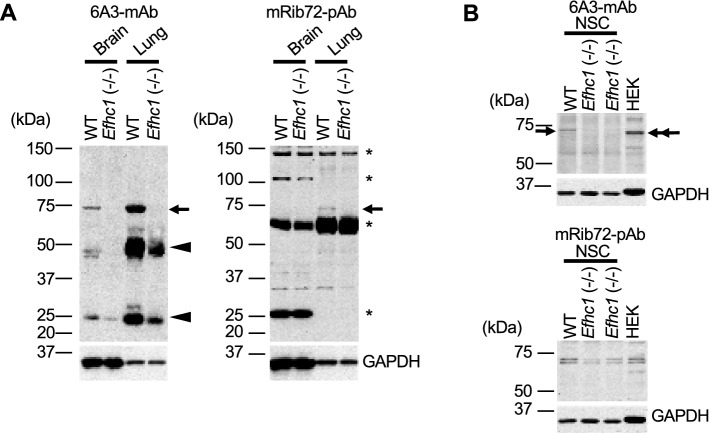
The 6A3-mAb specifically detects myoclonin1 at ~ 75 kDa, while mRib72-pAb detects multiple non-specific signals besides myoclonin1. (**A**) Western blots of brain and lung lysates probed with 6A3-mAb or mRib72-pAb (two independent experiments,* N* = 1 WT and 1 *Efhc1*^−/−^). Myoclonin1 was detected by both antibodies at the expected size (~ 75 kDa, arrows) in the lysates from WT, but not in those of *Efhc1*^−/−^ mouse. The 75 kDa band by mRib72-pAb was quite weaker than that of 6A3-mAb in the lung, and it was hardly detectable in the brain. The mRib72-pAb also detected additional bands (asterisks) that are much more intense than that of myoclonin1, and those remained in *Efhc1*^−/−^ mouse. Mouse IgG in mouse tissue lysates was detected by anti-mouse IgG secondary antibody (arrow heads in left panel). (**B**) Western blots of lysates from NSC and HEK cultured cells (two independent experiments,* N* = 1 WT and 2 *Efhc1*^−/−^). The 6A3-mAb detected 75 kDa band in cultured mouse neurosphere cells (NSC, arrow) from WT and HEK cells (double arrow), and this band well disappeared in *Efhc1*^−/−^ mouse. The molecular size of human myoclonin1 (640 amino acids, a.a.) in HEK cells is a little smaller than that of mouse myoclonin1 (648 a.a., GenBank accession number: ACB20692). In contrast, the mRib72-pAb detected multiple bands those remained in *Efhc1*^−/−^ mouse. An antibody to GAPDH was used as a control and shown in the lower panels (**A**, **B**).

To verify the specificity of 6A3-mAb monoclonal and mRib72-pAb polyclonal antibodies, we at first performed western blot analyses. The 6A3-mAb successfully detected a 75 kDa band of myoclonin1 in brain and lung tissue lysates of wild-type (WT) mouse, and these bands well disappeared in *Efhc1*^−/−^ mouse (Fig. 1A—left and Supplementary Fig. S1). The 6A3-mAb did not show apparent extra bands. In contrast, although mRib72-pAb was able to detect the myoclonin1 band in lung which disappeared in that of *Efhc1*^−/−^ mouse, mRib72-pAb detected additional bands with much higher intensities than that of myoclonin1 and these bands remained in *Efhc1*^−/−^ mouse (Fig. 1A—right). The mRib72-pAb hardly detected the 75 kDa band of myoclonin1 in brain of WT mice, possibly because of low sensitivity (Fig. 1A—right). Previous studies^14–16^ showed immunosignals of mRib72-pAb in mitotic apparatuses such as mitotic spindle and midbody in cultured cells including mouse neurosphere cells (NSC) and human embryonic kidney (HEK) cells. We re-investigated whether mRib72-pAb and 6A3-mAb can specifically detect myoclonin1 in NSC and HEK cell cultures. In western blot analyses of NSC cell lysates, 6A3-mAb again successfully detected the 75 kDa band in WT mouse, and the band well disappeared in *Efhc1*^−/−^ mouse (Fig. 1B—top). In contrast, mRib72-pAb detected multiple sized bands in both WT and *Efhc1*^−/−^ mice (Fig. 1B—bottom). In order to investigate the nature of proteins recognized by mRib72-pAb, we further performed two-dimensional (2-D) gel electrophoresis of brain lysate from *Efhc1*^−/−^ mouse followed by western blot analysis with mRib72-pAb and peptide mass fingerprinting with liquid chromatography electrospray ionization tandem mass spectrometry (LC–ESI–MS/MS) (Supplementary Figs. S2 and S3). The 2-D gel analysis showed 2 spots, 26 and 60 kDa, in very high intensities (Supplementary Fig. S2A—top). The LC–ESI–MS/MS revealed that these 26 and 60 kDa proteins were not myoclonin1 but glutathione S-transferase Mu 1 and dihydrolipoamide S-acetyltransferase precursor, respectively (Table 1**)**. As a control, Spot 3 (37 kDa) was detected by anti-glyceraldehyde-3-phosphate dehydrogenase (GAPDH) antibody (Supplementary Fig. S2A—middle), and confirmed to be GAPDH by LC–ESI–MS/MS (Table 1). These results indicate that mRib72-pAb crossreacts with non-myoclonin1 proteins with high affinities.

**Table 1:** mRib72 immunoreactive proteins were not myoclonin1 isoforms.

Spot #	Identified proteins	NCBI GI number	Nominal mass (*M*r)	Calculated p*I*	Sequence coverage (%)	MASCOT score ^a)^	Number of identified peptides
1	Glutathione S-transferase Mu 1	gi|6754084	26,067	7.71	58	837	19
2	dihydrolipoamide S-acetyltransferase precursor	gi|16580128	59,389	5.71	43	1561	39
3	Glyceraldehyde-3-phosphate dehydrogenase	gi|55153885	36,093	7.59	56	1158	55

^a^MASCOT score is defined as − 10 × Log(*P*), where *P* is the probability that the observed match is a random event. Scores greater than 67 are significant (*p* < 0.05).

### Myoclonin1 is not expressed in neurons but cells with motile cilia

**Figure 2:**
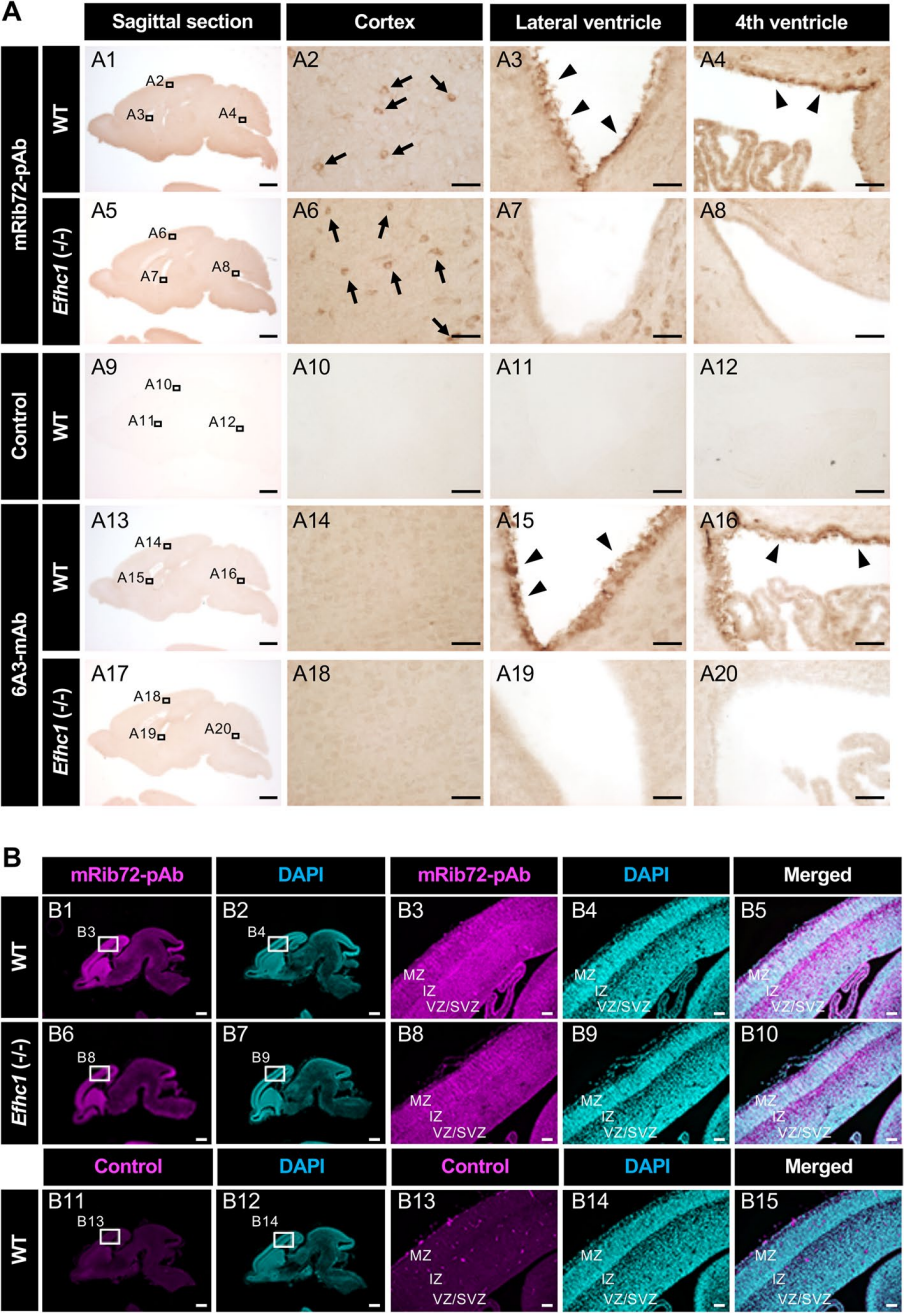
The mRib72-pAb, but not 6A3-mAb, shows immunosignals in neurons that remained in *Efhc1*^−/−^ mouse. (**A**) Sagittal brain sections from postnatal day 14 (P14) WT and *Efhc1*^−/−^ mice were DAB-stained with mRib72-pAb (top two rows) or 6A3-mAb (bottom two rows) (two independent experiments,* N* = 1 WT and 1 *Efhc1*^−/−^). The mRib72-pAb showed immunosignals in neurons at cerebral cortex (arrows) in WT as reported previously^14,17^, but these signals remained in *Efhc1*^−/−^ mouse. The 6A3-mAb did not show signals in neurons. Both mRib72-pAb and 6A3-mAb revealed signals at ependymal motile cilia (arrow heads), and those well disappeared in *Efhc1*^−/−^ mouse. The normal rabbit IgG was used as a negative control (middle row). A2–A4, A6–A8, A10–A12, A14–16 and A18–20: magnified images outlined in A1, A5, A9, A13 and A17, respectively. (**B**) Sagittal brain sections obtained from E16.5 WT and *Efhc1*^−/−^ mice were stained with the mRib72-pAb (magenta) and DAPI (cyan) (two independent experiments,* N* = 1 WT and 1 *Efhc1*^−/−^). The mRib72-pAb immunosignals were observed in neurons at cerebral cortex from both WT and *Efhc1*^−/−^ mice. The normal rabbit IgG was used as a negative control (bottom row). B3, B4, B8, B9, B13 and B14: magnified images outlined in B1, B2, B6, B7, B11 and B12, respectively. B5, B10 and B15: merged images of B3–B4, B8–B9 and B13–B14, respectively. Scale bars = 1 mm (A; Sagittal section), 50 μm (**A**; Cortex, Lateral ventricle, and 4th ventricle), 500 μm (**B**; Sagittal section) and 50 μm (**B**; high-magnification images). *VZ/SVZ* ventricular zone/sub ventricular zone, *IZ* intermediate zone, *MZ* marginal zone.

Previous studies^14,17^ showed that mRib72-pAb immunosignals in neurons. Our immunohistochemistry on pre- and postnatal mouse brain sections also showed mRib72-pAb signals in cortical neurons from WT, but those signals remained in *Efhc1*^−/−^ mouse (Fig. 2A, B). In contrast, 6A3-mAb did not show any signals in cerebral cortex in both mice (Fig. 2A). Meanwhile, both mRib72-pAb and 6A3-mAb showed intense signals at the ependymal motile cilia in WT and these signals well disappeared in *Efhc1*^−/−^ mouse (Fig. 2A), the observations are consistent to our previous study^10^. These results indicate that both mRib72-pAb and 6A3-mAb well detects myoclonin1 at ependymal motile cilia, but the mRib72-pAb immunosignals in neurons are non-specific.

### Myoclonin1 does not localize at mitotic apparatuses

**Figure 3:**
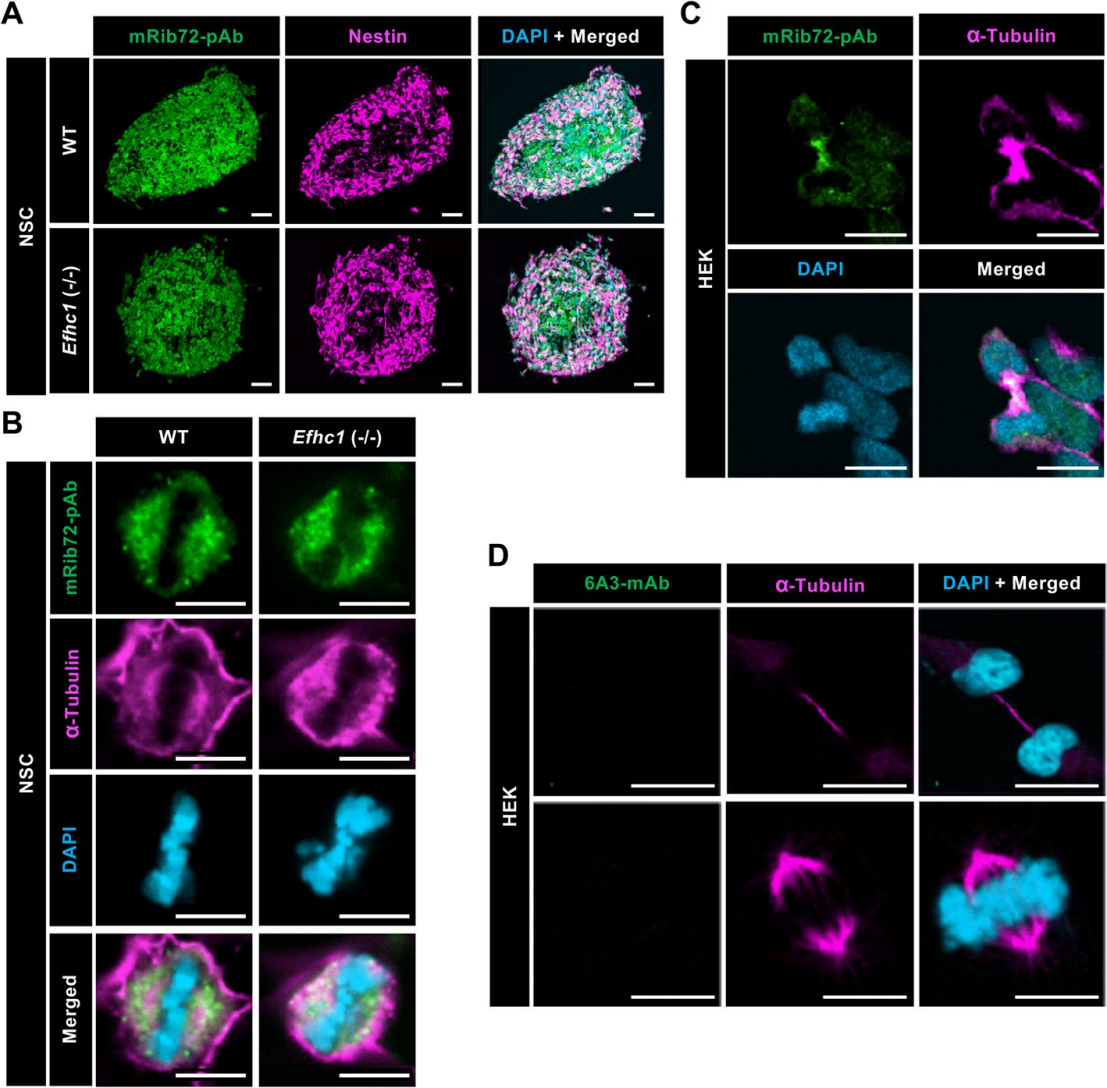
The mRib72-pAb, but not 6A3-mAb, shows immunosignals at mitotic apparatuses that remained in *Efhc1*^−/−^ mouse. (**A**) NSC derived from WT and *Efhc1*^−/−^ mouse brains at E14.5 were stained with mRib72-pAb (green), antibody to Nestin (magenta) and DAPI (cyan) (two independent experiments,* N* = 1 WT and 2 *Efhc1*^−/−^). Signals of mRib72-pAb were observed in cytoplasm of NSC from both WT and *Efhc1*^−/−^ mice. *N* = 3 WT and 4 *Efhc1*^−/−^ spheres. (**B**) During cellular mitosis, NSC were stained with mRib72-pAb (green), antibody to α-tubulin (magenta) and DAPI (cyan). Signals of mRib72-pAb were observed in mitotic spindle in cells from WT as reported previously^14^, but remained in *Efhc1*^−/−^. *N* = 13 WT and 6 *Efhc1*^−/−^ cells. (**C**) HEK cells were stained with mRib72-pAb (green), antibodies to α-tubulin (magenta) and DAPI (cyan) (two independent experiments). Midbody was stained with mRib72-pAb and α-tubulin. (**D**) HEK cells were stained with 6A3-mAb (green), antibody to α-tubulin (magenta) and DAPI (cyan) (two independent experiments). The 6A3-mAb did not show signals in midbody and mitotic spindles. Scale bars = 40 μm (**A**), 6 μm (**B**), and 10 μm (**C**, **D**).

As reported previously^14^, immunocytochemistry on cultured NSC from WT mouse, those were well positive for Nestin (marker for neural stem cells), showed that the mRib72-pAb surely developed immunosignals at cytoplasm (Fig. 3A) and at mitotic spindles during cellular mitosis (Fig. 3B). However, these signals remained in *Efhc1*^−/−^ (Fig. 3A, B). In dividing HEK cells, mRib72-pAb signals were also observed at the midbody (Fig. 3C) as reported in previous studies^14–16^, but 6A3-mAb did not show any signals at these mitotic apparatuses (Fig. 3D). These results indicate that myoclonin1 does not localize at mitotic apparatuses such as mitotic spindle and midbody.

### Myoclonin1 deficiency does not critically affect cortical development

**Figure 4:**
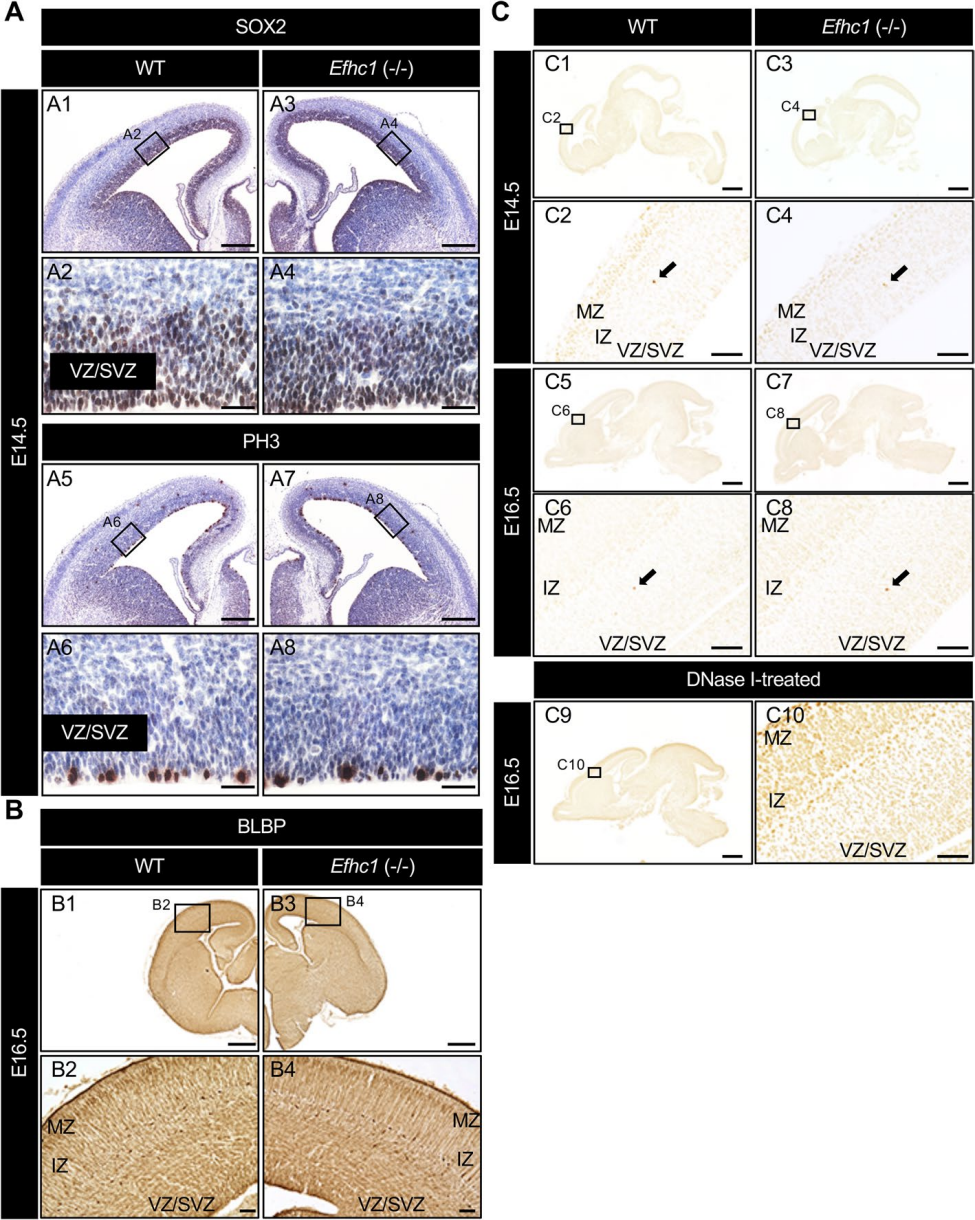
No visible abnormalities in cortical development of *Efhc1*^−/−^ mouse. (**A**) DAB-staining of coronal brain sections from E14.5 WT and *Efhc1*^−/−^ mice with antibodies to SOX2 or PH3 (two independent experiments, *N* = 3 WT and 3 *Efhc1*^−/−^). Nuclei were stained with hematoxylin (blue). SOX2 and PH3 expressions were observed at ventricular zone (VZ)/sub ventricular zone (SVZ). Note that there were no visible differences in their distributions between WT and *Efhc1*^−/−^. A2, A4, A6 and A8: magnified images outlined in A1, A3, A5 and A7, respectively. (**B**) Coronal brain sections from E16.5 WT and *Efhc1*^−/−^ mice were DAB-stained with antibody to BLBP (two independent experiments,* N* = 1 WT and 1 *Efhc1*^−/−^). No obvious difference of radial glia process extension was observed between WT and *Efhc1*^−/−^. B2 and B4: magnified images outlined in B1 and B3, respectively. (**C**) TUNEL assay on the sagittal brain sections of E14.5 and E16.5 WT and *Efhc1*^−/−^ mice (two independent experiments,* N* = 2 WT and 2 *Efhc1*^−/−^ for E14.5; *N* = 1 WT and 1 *Efhc1*^−/−^ for E16.5). DNase I-treated section was used as a positive control. There were no differences in the TUNEL positive cell numbers between WT and *Efhc1*^−/−^ mice in both stages. C2, C4, C6, C8 and C10: magnified images outlined in C1, C3, C5, C7 and C9, respectively. Scale bars = 200 μm (**A**; low-magnification images), 25 μm (**A**; high -magnification images), 500 μm (B1, B3, C1, C3, C5, C7 and C9), 50 μm (B2, B4, C2, C4, C6, C8 and C10). *IZ* intermediate zone, *MZ* marginal zone.

The Belgium group reported that a suppression of *Efhc1* by shRNA-mediated RNAi in rat embryonic brain caused disruption of mitotic spindle structure, increased apoptosis, impaired locomotion of postmitotic neurons, and marked disruption of radial migration^17^. We investigated whether *Efhc1*^−/−^ mouse has any abnormalities in cerebrocortical progenitors, locomotion of postmitotic neurons, or radial glia scaffold organization by using antibodies for SOX2 (a marker for progenitor cells), phospho-Histone H3 (PH3; a marker for mitotic cells), and brain lipid-binding protein (BLBP; a marker for radial glia) those were used in the previous study^17^. We did not observe any apparent differences in the distribution of immunopositive cells detected by these antibodies between WT and *Efhc1*^−/−^ mice (Fig. 4A, B). We also performed TUNEL assay on brain sections, however it revealed no differences between WT and *Efhc1*^−/−^ (Fig. 4C). These results indicate that the elimination of myoclonin1 does not largely affect cell cycle exit of cerebral cortical progenitors, radial glia scaffold organization and apoptosis.

## Discussion

The present study described a re-evaluation of expression profile of myoclonin1 and the discrepancy in histological phenotypes between the *Efhc1* constitutive knock-out in mice^10,12^ and acute shRNA-mediated knock-down in rats^17^. We confirmed that myoclonin1 is predominantly expressed in ependymal motile cilia but not in neurons of brain. These present and previous results^10,12^ are consistent to the previous in situ hybridization analysis showing that *Efhc1* mRNA was predominantly appeared in the ependymal and choroid plexus epithelial cells but not in neurons in mouse and rat brains^13^. We also showed that myoclonin1 is not expressed in mitotic apparatuses such as mitotic spindles and midbody in dividing cells, and that the complete elimination of myoclonin1 did not critically affect cell division and migration of neurons in the cerebral cortex of *Efhc1*^−/−^ mouse. These phenotypic discrepancy between the rat with acute knock-down of *Efhc1* by shRNA with drastic alterations in cell division and neuronal migration in embryonic cerebral cortex^17^ and the *Efhc1*^−/−^ mouse without those alterations in present and previous studies^12^ is most possibly explained by the off-target effect of shRNA^18^. Our present results therefore deny the proposal of the Belgium group that myoclonin1 is expressed in neurons, radial glia cells, mitotic apparatuses, and plays critical roles in neuronal cell division and migration during cortical development^14–17^. Our present and previous studies^10,12,13^ may also deny our own previous proposal that the interaction of myoclonin1 and R-type voltage-dependent Ca^2+^ channel (Ca_v_2.3) in neurons plays a role in JME^1^. Meanwhile, our another proposal that the functional interaction of myoclonin1 and the transient receptor potential M2 channel (TRPM2) plays a role in the pathogenesis of JME^19^ may still survive as a possible pathomechanism of JME because of the TRPM2 expression in ependymal cells in addition to neurons.

Recently we identified ciliogenesis associated kinase 1 (*CILK1*), also known as intestinal-cell kinase (*ICK*), as another gene responsible for JME^20^. Interestingly, CILK1 is again highly expressed in choroid plexus and ependymal cells^20^. *PRICKLE1* and *PRICKLE2* gene mutations have been identified in patients with JME and other types of myoclonic epilepsies^21,22^, and disruptions of these genes in zebrafish, *D. melanogaster* or mouse lead to increased seizure susceptibility^22,23^. Interestingly again, these genes are correlated to ciliogenesis or ciliary functions^24–26^. These observations further support the notion that impairments of cells with motile cilia in brain cause JME.

*Efhc1*^−/−^ mice developed frequent spontaneous myoclonus, decreased seizure threshold, and reduced ciliary beating frequencies (CBF) of postnatal ependymal motile cilia^12^ as well as neonatal choroid plexus epithelial cilia^27^. Although the reduced CBF in *Efhc1*^−/−^ mice may not be directly relevant to the pathogenesis of JME which has been inferred from the inconsistency of seizure susceptibility and CBF reduction in heterozygous *Efhc1*^+*/−*^ mice^12^, other possible impairments of motile cilia, ependymal cells or choroid plexus (e.g. sensory antenna, ion exchange, cerebral spinal fluid (CSF) secretion, or pH of CSF, etc.) could be the causes of JME. Further studies are warranted to figure these out.

In conclusion, our results presented here indicate that myoclonin1 is not expressed in neurons, not a regulator of cell division or neuronal migration during cortical development, but expressed in cells with motile cilia in brain and therefore suggest that *EFHC1*-dependent JME is a motile ciliopathy.

## Materials and methods

### Animal experiments

All animal experimental protocols were approved by the Animal Experiment Committee of Institute of Physical and Chemical Research (RIKEN). All animal breeding and experimental procedures were performed in accordance with the ARRIVE guidelines and the guidelines of the Animal Experiments Committee of RIKEN. Animals were maintained on 12 h light/dark cycle with ad libitum access to food and water at the Research Resources Division (RRD) of the RIKEN Center for Brain Science. *Efhc1*-deficient mouse was described previously^12^. The heterozygous mice were maintained on the C57BL/6J (B6J) background, and the resultant heterozygous mice were interbred to obtain WT, heterozygous, and homozygous mice. Genotyping was carried out as described previously^12^.

### Western blot analysis

Brain and lung (*N* = 1 WT and 1 *Efhc1*^−/−^) samples, cultured NSC (*N* = 1 WT and 2 *Efhc1*^−/−^) and HEK cells were homogenized in ice-cold 1X phosphate-buffered saline (PBS) supplemented with protease inhibitors (Complete, Roche). The following primary antibodies were used: mouse monoclonal anti-myoclonin1 (6A3-mAb, 1:2000 dilution) or rabbit polyclonal anti-myoclonin1 (mRib72-pAb, 1:200 dilution; kind gift from Prof. Ritsu Kamiya, University of Tokyo, Japan). HRP-conjugated anti-mouse IgG (W402B, Promega, 1:5000 dilution) or anti-rabbit IgG (sc-2004, SANTA CRUZ, 1:2000 dilution) were used for secondary antibody. Labeled proteins were revealed by using enhanced chemiluminescence (ECL) detection (Perkin-Elmer). Membranes were then washed with Restore Plus Western Blot stripping buffer (Thermo Scientific), re-probed with rabbit polyclonal anti-GAPDH (sc-25778, SANTA CRUZ, 1:1000 dilution) and HRP-conjugated anti-rabbit IgG antibody and revealed as described above.

### 2-D electrophoresis

For 2-D electrophoresis, mouse brains at 3-month-old from *Efhc1*^−/−^ mice (*N* = 3) were homogenized in 8 M Urea, 2% CHAPS, 2% Dithiothreitol (DTT), 1% IPG buffer, pH 3–10 NL (GE Healthcare), and Bromophenol blue (BPB). First dimensional isoelectric focusing (IEF) was carried out on an Immobiline DryStrip pH3-10NL, 7 cm (GE Healthcare) using an Ettan IPGphor (GE Healthcare). Each strip was rehydrated for 12 h with sample lysate (0.1 µg). Isoelectric focusing (IEF) was then carried out. Strips were subjected to a two-step equilibration (6 M Urea, 2% SDS, 30% glycerol, BPB) in 0.5% DTT and 4.5% iodoacetamide (nacalai tesque) buffers before proceeding to SDS-PAGE. Proteins were separated for the second dimension on 5–20% gradient SDS–polyacrylamide gel (Super Sep Ace, Wako pure reagents). After 2-D electrophoresis was terminated, one gel was stained in a solution containing 0.1% Coomassie Brilliant Blue-R250 (CBB) 10% methanol and 0.5% acetic acid, and then destained in a solution containing 10% methanol and 0.5% acetic acid. The other one gel was used for western blot analysis with mRib72-pAb.

### In-gel digestion for LC–ESI–MS/MS and mass analysis

Spots corresponding to the immunosignals of the mRib72-pAb were excised from CBB stained gels. The gel pieces were washed out three times with water for 30 s at 37 °C. Gels were then destained with 50 mM NH_4_HCO_3_ (09830, Fluka)/50% CH_3_CN (34967, Fluka) at 37 °C for 10 min, dehydrated with 50 µL CH_3_CN for 10 min at 37 °C, and dried completely in a vacuum centrifuge. Gel pieces were reduced with 50 µL 0.01 M DTT (D5545, SIGMA)/100 mM NH_4_HCO_3_ for 15 min at 50 °C, and incubated with 2 µL of 0.25 M IAA (I1149, SIGMA)/100 mM NH_4_HCO_3_ for 15 min at dark place. The gels were washed with 100 mM NH_4_HCO_3_, and 50 mM NH_4_HCO_3_/50% CH_3_CN, and dried completely in a vacuum centrifuge. The samples were incubated at 4 °C for 15 min in 10 ng/μL Sequencing Grade Modified trypsin (V5111, Promega) solution, and digested at 37 °C for 12 h. Peptides were extracted from the gel pieces with 50% CH_3_CN/1% TFA (208-02741, WAKO pure reagents), and dried completely in a vacuum centrifuge. The samples (*N* = 3, each spot) were dissolved in 2% CH_3_CN/0.1% TFA, and subjected to LC–MS/MS using LTQ linear ion trap mass spectrometer (Thermo Fisher Scientific) at the Support Unit for Bio-Material Analysis in RIKEN CBS Research Resources Division. Mascot search engine^28^ (ver. 2, Matrix Science; http://www.matrixscience.com/search_form_select.html) was used for searching mouse proteins in the NCBInr 20110521 (14141183 sequences; 4845787524 residues) database.

### Primary neurosphere culture

The dorsal telencephalons of embryonic WT and *Efhc1*^−/−^ mice (*N* = 1 WT and 2 *Efhc1*^−/−^) at day 14.5 were mechanically dissociated and incubated in trypsin solution at 37 °C for 5 min. Cells were resuspended in Neurobasal medium (Thermo Fisher Scientific) containing N2 and B27 supplements (Thermo Fisher Scientific), 100 ng/mL epidermal growth factor (EGF, Sigma) and 10 ng/mL basic fibroblast growth factor (bFGF, Sigma) and filtered through a 40 µm nylon mesh. The resulting cell suspension of 1 × 10^6^ cells were transferred to non-adherent T25 culture flask and cultured as suspension for 4 days. Neurosphere cells were transferred onto glass coverslip coated with Poly-D-Lysine/Laminin (Corning) in wells of 24 well plate.

### Immunocytochemistry and immunohistochemistry

Preparation of fixed cells, paraffin (6-µm-thick) sections of mouse brain (*N* = 3 WT and 3 *Efhc1*^−/−^ for E14.5; *N* = 2 WT and 2 *Efhc1*^−/−^ for E16.5; and *N* = 1 WT and 1 *Efhc1*^−/−^ for P14), immunocytochemical and immunohistochemical analyses were done as previously^10^. For staining using mouse primary antibodies, the Mouse on Mouse (M.O.M.) detection kit (BMK-2202, VECTOR Laboratories) was used to reduce endogenous mouse IgG staining. The following primary antibodies were used: mouse monoclonal anti-myoclonin1 (6A3-mAb, 1:1000 dilution), rabbit polyclonal anti-myoclonin1 (mRib72-pAb, 1:50 dilution), mouse monoclonal anti-Nestin antibody (MAB353, Millipore, 1:400 dilution), mouse monoclonal α-Tubulin antibody (T9026, SIGMA, 1:500 dilution), rabbit polyclonal anti-SOX2 antibody (AB5603, Millipore, 1:1000 dilution), rabbit monoclonal phospho-Histone H3 (PH3, #04-746, Millipore, 1:2000 dilution), or rabbit polyclonal anti-BLBP (ab32423, abcam, 1:2000 dilution). Biotin conjugated anti-mouse IgG or anti-rabbit IgG (VECTOR Laboratories, 1:200 dilution) were used for secondary antibody. Immunoreactivity was visualized by using a Vectastain Elite ABC kit (VECTOR Laboratories), developed by using the ImmPACT 3,3′-diaminobenzidine (DAB) Peroxidase (HRP) Substrate kit. For fluorescent immunocytochemical and immunohistochemical analyses, Alexa Fluor 488-, 594- or 647 conjugated anti-mouse IgG or anti-rabbit IgG (Thermo Fisher Scientific, 1:400 dilution) were used for secondary antibody. Nuclei were stained with DAPI. The normal mouse and rabbit IgGs (Santa Cruz Biotechnology) were used as negative controls. Images were acquired by the TCS SP2 (Leica), the AX80 (Olympus) or Biozero BZ-X710 (KEYENCE) microscope.

### TUNEL assay

Paraffin (6-μm-thick) sections of mouse brains (*N* = 2 WT and 2 *Efhc1*^−/−^ for E14.5; *N* = 1 WT and 1 *Efhc1*^−/−^ for E16.5) were used in the assay. Apoptotic cells in paraffin sections were detected by using the DeadEnd Colorimetric TUNEL System (Promega). For a negative control, sections were incubated in buffer without the recombinant terminal deoxynucleotidyl transferase (rTdT) enzyme. DNase I-treated sections were used as positive controls. Images were acquired by the Biozero BZ-X710 (KEYENCE) microscope.

## Supplementary Information

Supplementary Information.

## Data Availability

All data generated or analyzed during this study are included in this published article and its Supplementary Information File.
