# Supplementary material for: Epilepsy protein Efhc1/myoclonin1 is expressed in cells with motile cilia but not in neurons or mitotic apparatuses in brain
Source: Sci Rep. 2020 Dec 16;10:22076. doi: 10.1038/s41598-020-79202-4 (PMC7744795; doi:10.1038/s41598-020-79202-4)
Supplement: Supplementary file 1 — Supplementary Information. [file 41598_2020_79202_MOESM1_ESM.pdf]

Epilepsy protein Efhc1/myoclonin1 is expressed in cells with motile cilia but not in neurons or mitotic apparatuses in brain

(Supplementary Information)

Toshimitsu Suzuki[1,2], Ikuyo Inoue[2], and Kazuhiro Yamakawa[1,2*]

[1]Department of Neurodevelopmental Disorder Genetics, Institute of Brain Science, Nagoya City University Graduate School of Medical Science, Nagoya, Aichi, 467-8601, Japan.
[2]Laboratory for Neurogenetics, RIKEN Center for Brain Science, Wako, Saitama, 351-0198, Japan.

*Correspondence and requests for materials should be addressed to K.Y. (email: yamakawa@med.nagoya-cu.ac.jp).
Kazuhiro Yamakawa, Ph.D.,
Department of Neurodevelopmental Disorder Genetics, Institute of Brain Science, Nagoya City University Graduate School of Medical Science,
1 Kawasumi, Mizuho-cho, Mizuho-ku Nagoya, Aichi 467-8601, Japan,
Tel: +81-52-851-5612, Email: yamakawa@med.nagoya-cu.ac.jp

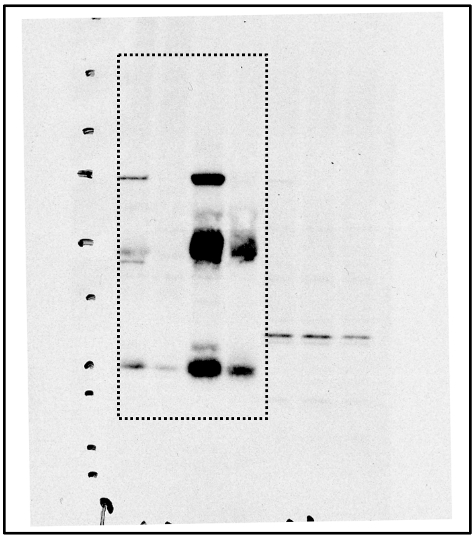

Figure 1A Left

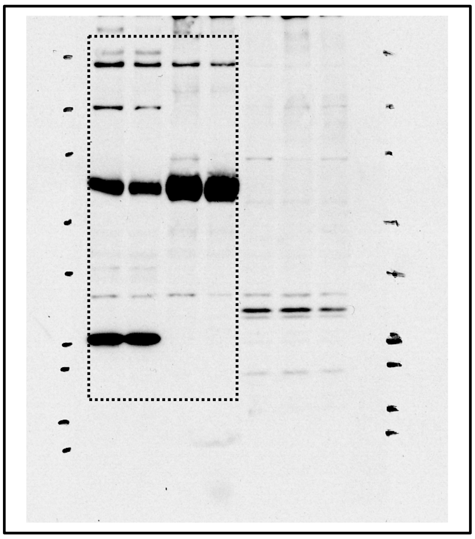

Figure 1A Right

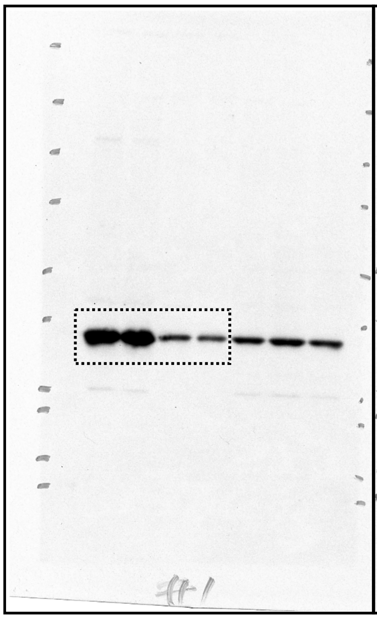

Figure 1A Left GAPDH

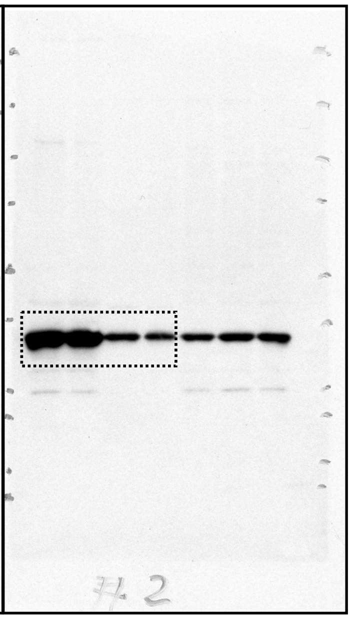

Figure 1A Right GAPDH

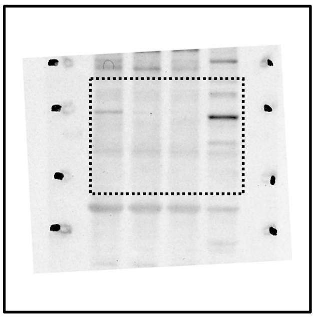

Figure 1B Top

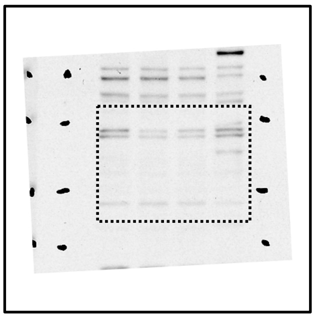

Figure 1B Bottom

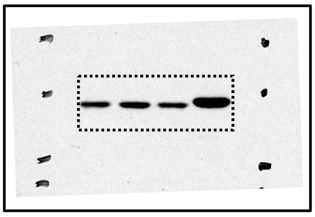

Figure 1B Top GAPDH

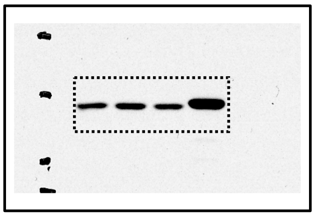

Figure 1B Bottom GAPDH

Supplementary Figure S1. Full size western blot images. Original scanned western blot data that were used to generate Figure 1. Dashed rectangles in the images indicate the locations of the cropped images.

A

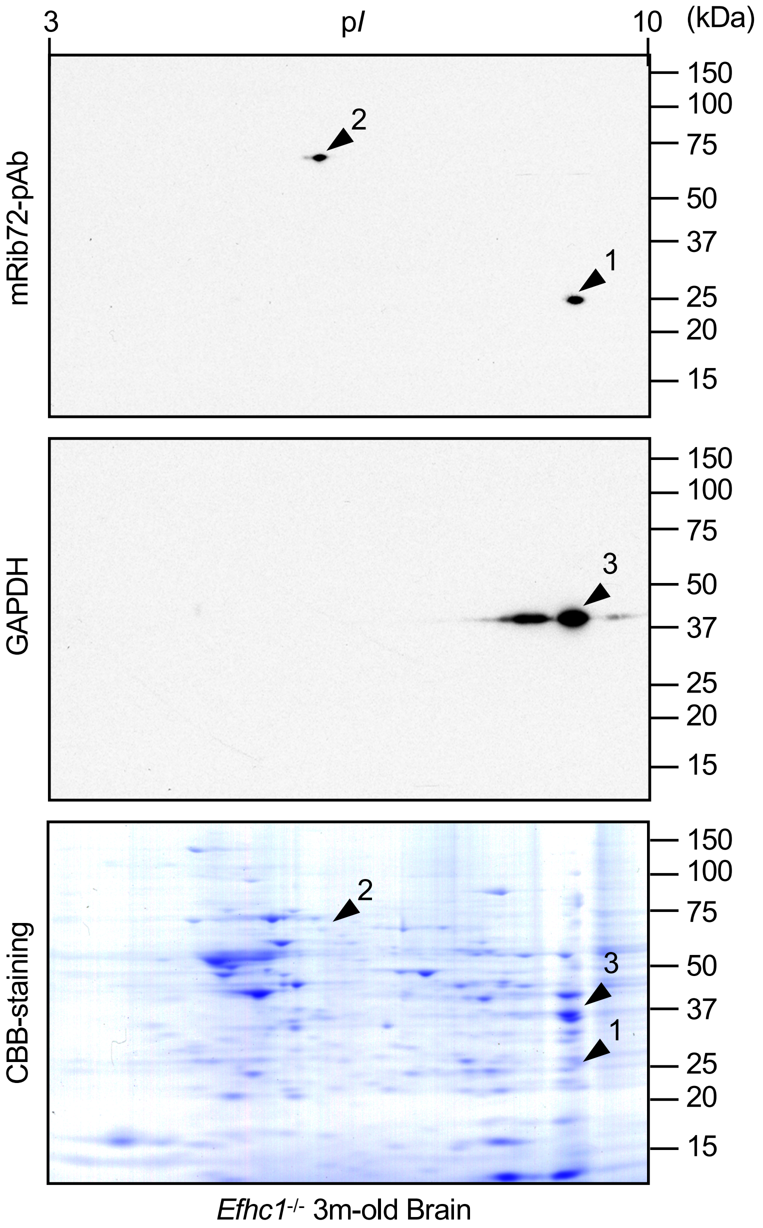

B

1 M**PMILGYWNV** **R**GLTHPIR**ML** **LEYTDSSYDE**
31 **KRYTMGDAPD** **FDR**SQWLNEK FK**LGLDFPNL**
61 **PYLIDGSHKI** **TQSNAILR**YL AR**KHHLDGET**
91 **EEERIRADIV** **ENQVMDTRMQ** **LIMLCYNPDF**
121 **EK**QKPEFLKT IPEKMKLYSE FLGK**RPWFAG**
151 **DK**VTYVDFLA YDILDQYRMF EPK**CLDAFPN**
181 **LR**DFLARFEG LKKISAYMKS SR**YIATPIFS**
211 **K**MAHWSNK

C

1 YYSLPPHQK**V** **PLPSLSPTMQ** **AGTIAR**WEKK
31 EGEKISEGDL IAEVETDKAT VGFESLEECY
61 MAKILVPEGT RDVPVGSIIC ITVEKPQDIE
91 AFKNYTLDLA AAAAPQAAPA AAPAPAAAPA
121 APSASAPGSS YPTHMQIVLP APSPTMTMGT
151 VQRWEKKVGE KLSEGDLLAE IETDKATIGF
181 EVQEEGYLAK IXVPEGTR**DV** **PLGAPXCIIV**
211 **EK**QEDIAAFA DYRPTEVTSL KPQAAPPAPP
241 PVAAVPPTPQ PVAPTPSAAP AGPKGR**VFVS**
271 **PLAK**KLAAEK **GIDLTQVK**GT GPEGRIIK**KD**
301 **IDSFVPSKAA** **PAAAAAMAPP** **GPRVAPAPAG**
331 **VFTDIPISNI** **R**RVIAQRLMQ SKQTIPHYYL
361 SVDVNMGEVL LVRKELNKML EGK**GKISVND**
391 **FIIK**ASALAC LK**VPEANSSW** **MDTVIRQNHV**
421 **VDVSVAVSTP** **AGLITPIVFN** **AHIKGLETIA**
451 **SDVVSLASK**A REGK**LQPHEF** **QGGTFTISNL**
481 **GMFGIKNFSA** **IINPPQACIL** **AIGASEDK**LI
511 PADNEK**GFDV** **ASVMSVTLSC** **DHRVVDGAVG**
541 **AQWLAEFKKY** **LEKPITMLL**

Supplementary Figure S2. The mRib72-pAb crossreacted with non-myoclonin1 proteins with high affinities. (**A**) The 2-D western blot of brain lysates from *Efhc1*-/- mouse at 3-month-old probed with mRib72-pAb (three independent experiments, $N = 3$ *Efhc1*-/-). The antibody revealed 2 distinct spots at 26 (spot 1) and 60 (spot 2) kDa (upper panel). An antibody to GAPDH was used as a control (middle panel). The 2-D gel was stained with Coomassie Brilliant Blue (CBB, lower panel). The spots 1, 2 and 3 were subjected to peptide-mass fingerprinting. (**B, C**) LC-ESI-MS/MS analysis and database search indicated that spot 1, 2 and 3 fitted to glutathione S-transferase Mu 1 (B), dihydrolipoamide S-acetyltransferase precursor (C), and GAPDH (not shown), respectively (three independent experiments, $N = 3$, each spot). Peptides detected by mass spectrometry were indicated in red boldface (B, C).

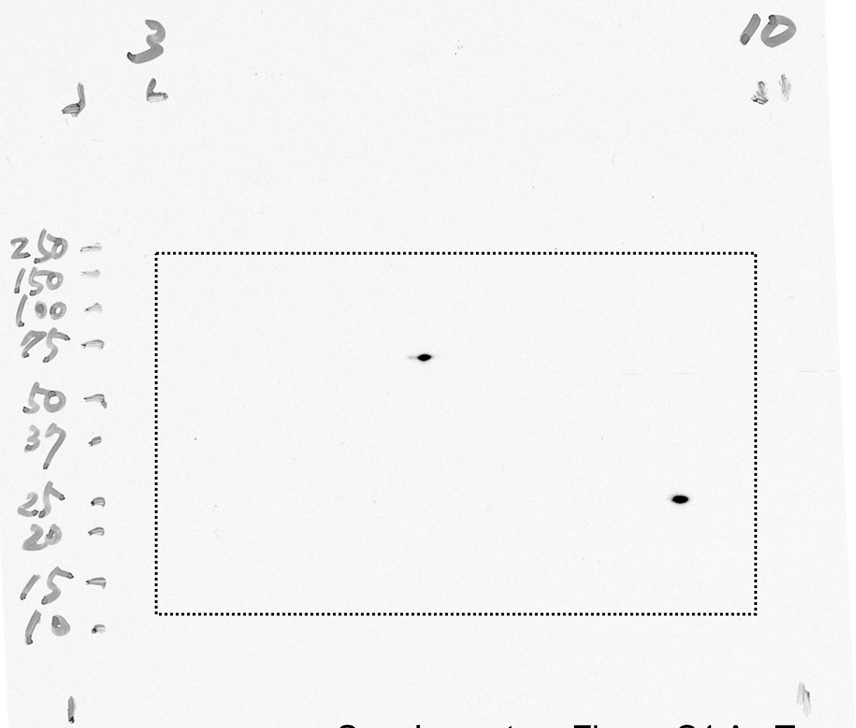

Supplementary Figure S1 A - Top

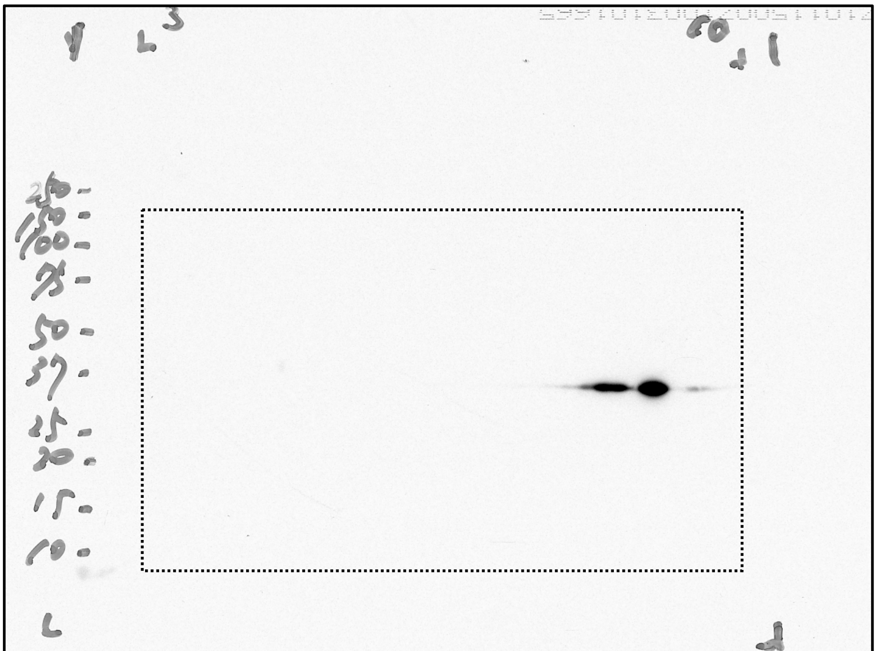

Supplementary Figure S1 A - Middle

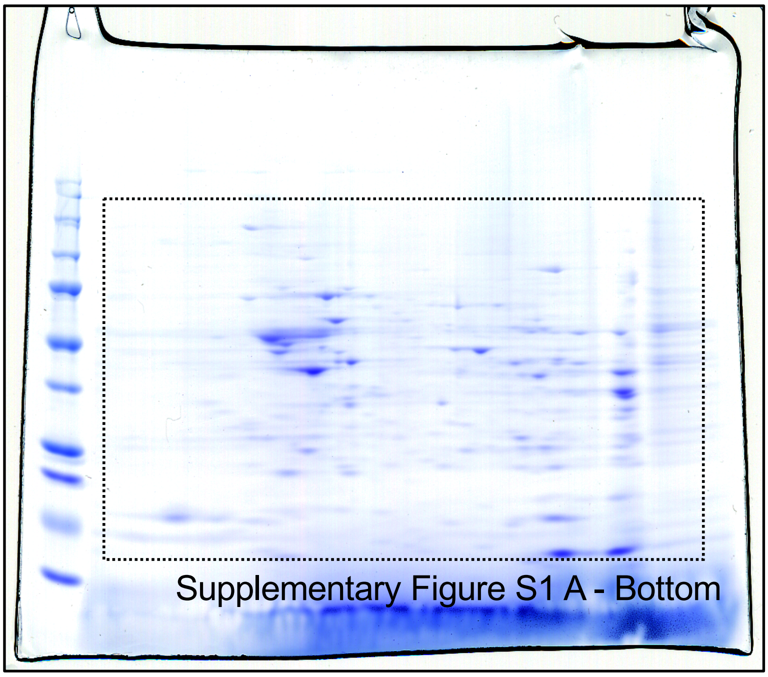

Supplementary Figure S1 A - Bottom

Supplementary Figure S3. Full size 2-D western blot and gel images. Original scanned western blot and gel data that were used to generate Supplementary Figure S2. Dashed rectangles in the images indicate the locations of the cropped images.
